# Predicting dynamic response to neoadjuvant chemotherapy in breast cancer: a novel metabolomics approach

**DOI:** 10.1002/1878-0261.13216

**Published:** 2022-04-14

**Authors:** Caridad Díaz, Carmen González‐Olmedo, Leticia Díaz‐Beltrán, José Camacho, Patricia Mena García, Ariadna Martín‐Blázquez, Mónica Fernández‐Navarro, Ana Laura Ortega‐Granados, Fernando Gálvez‐Montosa, Juan Antonio Marchal, Francisca Vicente, José Pérez del Palacio, Pedro Sánchez‐Rovira

**Affiliations:** ^1^ Fundación MEDINA Centro de Excelencia en Investigación de Medicamentos Innovadores en Andalucía Granada Spain; ^2^ Medical Oncology Unit University Hospital of Jaén Spain; ^3^ 16741 Department of Signal Theory, Networking and Communications University of Granada Spain; ^4^ 16741 Biopathology and Regenerative Medicine Institute (IBIMER) Centre for Biomedical Research University of Granada Spain; ^5^ 16741 Instituto de Investigación Biosanitaria ibs.GRANADA University of Granada Spain; ^6^ 16741 Department of Human Anatomy and Embryology Faculty of Medicine University of Granada Spain; ^7^ 16741 Excellence Research Unit "Modeling Nature" (MNat) University of Granada Spain

**Keywords:** ASCA, breast cancer, LC‐HRMS, neoadjuvant chemotherapy, personalized medicine, treatment response

## Abstract

Neoadjuvant chemotherapy (NACT) outcomes vary according to breast cancer (BC) subtype. Since pathologic complete response is one of the most important target endpoints of NACT, further investigation of NACT outcomes in BC is crucial. Thus, identifying sensitive and specific predictors of treatment response for each phenotype would enable early detection of chemoresistance and residual disease, decreasing exposures to ineffective therapies and enhancing overall survival rates. We used liquid chromatography−high‐resolution mass spectrometry (LC‐HRMS)‐based untargeted metabolomics to detect molecular changes in plasma of three different BC subtypes following the same NACT regimen, with the aim of searching for potential predictors of response. The metabolomics data set was analyzed by combining univariate and multivariate statistical strategies. By using ANOVA–simultaneous component analysis (ASCA), we were able to determine the prognostic value of potential biomarker candidates of response to NACT in the triple‐negative (TN) subtype. Higher concentrations of docosahexaenoic acid and secondary bile acids were found at basal and presurgery samples, respectively, in the responders group. In addition, the glycohyocholic and glycodeoxycholic acids were able to classify TN patients according to response to treatment and overall survival with an area under the curve model > 0.77. In relation to luminal B (LB) and HER2+ subjects, it should be noted that significant differences were related to time and individual factors. Specifically, tryptophan was identified to be decreased over time in HER2+ patients, whereas LysoPE (22:6) appeared to be increased, but could not be associated with response to NACT. Therefore, the combination of untargeted‐based metabolomics along with longitudinal statistical approaches may represent a very useful tool for the improvement of treatment and in administering a more personalized BC follow‐up in the clinical practice.

## Graphical abstract

Recently, metabolomics emerged as a novel approach that may unravel the molecular factors conferring chemotherapy resistance in breast cancer. We aimed to decipher molecular profiles related with response to neoadjuvant chemotherapy in luminal B, triple‐negative, and HER2+ breast cancer subtypes. Interestingly, by combining untargeted metabolomics with ASCA, significant candidate metabolites were detected in response to neoadjuvant chemotherapy in the triple‐negative phenotype.

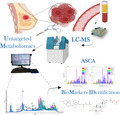

**Abbreviations**
AcN: acetonitrile
ASCA: ANOVA–simultaneous component analysis
AUC: area under the curve
BC: breast cancer
BMI: body mass index
CCs: cancer cells
DHA: docosahexaenoic acid
ER: estrogen receptors
FC: fold change
FDR: false discovery rate
FISH: fluorescent in situ hybridization
GDCA: glycodeoxycholic acid
GHCA: glycohyocholic acid
HER2: human epidermal growth factor 2
IDA: information dependent acquisition
Kyn: kynurenine
LB: luminal B
LC‐HRMS: liquid chromatography−high‐resolution mass spectrometry
MP: Miller and Payne
MVA: multivariate analysis
NACT: neoadjuvant chemotherapy
NR: nonresponders
PCA: principal component analysis
pCR: pathological complete response
PL: phospholipids
PR: progesterone receptors
QC: quality control
R: responders
ROC: receiver‐operating characteristic
RP: reverse phase
RSD: relative standard deviation
SVM‐linear: linear kernel support vector machine
t1: time 1
t2: time 2
t3: time 3
TN: triple negative
TNM: tumor nodes metastasis
TOF: time of flight
Trp: tryptophan
UVA: univariate analysis

## 1 Introduction

Breast cancer (BC) incidence continues rising, being the leading cause of cancer death in women in the last Global Cancer Statistics 2020 [1]. Resistance to chemotherapeutic drugs is still the main obstacle for any cancer treatment. Some cancer cells (CCs) have innate chemotherapy resistance while others acquire it during exposure. Thus, pathological nonresponse to the chemo agents facilitates tumor cell survival and uncontrolled proliferation or metastasis after treatment administration [2, 3, 4].

Nowadays, undergoing surgery after a successive combination of drugs is considered the gold standard for assessing tumor response [5, 6]. However, not all BC patients benefit from the neoadjuvant chemotherapy (NACT) setting and, therefore, it is critical to differentiate between the subjects that will respond positively and those who will not, in order to choose alternative and more effective therapies. Regarding NACT efficacy, recent studies tackle the relationship between BC phenotypes and treatment outcomes [7, 8, 9], revealing pathological complete response (pCR) as a surrogate biomarker of response and survival [10, 11]. Nevertheless, this procedure is invasive and time‐consuming. Thus, faster, less invasive and more sensitive tools are required in order to detect useful molecular and/or clinical predictors of pCR [12, 13].

On this point, metabolomics has quickly risen up as a novel approach in the cancer biomarker field for overcoming the current limitations of standard diagnostic and prognostic techniques [14, 15]. This expanding research area, combined with high‐throughput screening technologies, may help to unravel the subjacent molecular factors conferring true chemosensitivity to tumor recurrence, yet unknown. Indeed, it appears as the ‐omics science that better reflects the complex interactions from the genome expression to the phenotypic variations. Common metabolites directly or indirectly involved in the biology of cancer may serve as disease evaluators in group of patients. Several studies have already been conducted to explore the possibility of using panels of metabolites as biomarkers for early diagnosis and tumor characterization [16, 17, 18, 19, 20, 21, 22]. The abnormally accumulated metabolites derived from disrupted cancer metabolic pathways are newly described as oncometabolites, for example, D2‐hydroxyglutarate has an important function in prognosis and diagnosis of breast cancer and leukemia patients [23, 24, 25]. Thus, although detection of metabolic markers with an important role in oncological processes is appearing, research focused on finding discriminant biomarkers of NACT response in BC, and therefore, clinical outcome prognosis, is still sparse [12, 26, 27, 28]. Notably, the development of metabolic fingerprinting to find a molecular pattern that might predict chemoresistance depending on the molecular BC subtype would support the evidence for its use in the clinical practice.

Large‐scale data sets resulting from the untargeted metabolomics approach, in combination with other factors, such as time, are becoming increasingly intricate to analyze, and the use of traditional biostatistical methods cannot be applied straightforwardly to extract clear and definite results. Hence, the incorporation of advanced methods such as ANOVA–simultaneous component analysis (ASCA) has become crucial for understanding the complexity and heterogeneity of biological information. ASCA is a direct generalization of the analysis of variance for univariate data applied to the multivariate case [29, 30]. In consequence, longitudinal intervention studies over time, combined with untargeted metabolomics, may arise as an essential type of experimental approach in BC clinical research for discovering highly accurate markers or proven targets for tailored therapeutic treatments, detected in plasma of individuals with this disease [12, 30, 31]. However, to date, the definition of best practices for the analysis and interpretation of longitudinal metabolomics data is still a matter of research [32].

With this aim in view, here we explore whether untargeted metabolomics is able to determine molecular profiles of prediction to NACT response in a follow‐up of 92 BC patients with different phenotypes, integrating univariate analysis and ASCA. Grounded in a liquid chromatography–high‐resolution mass spectrometry (LC‐HRMS) platform‐based metabolomics analysis, plasma samples were studied at three different time points. Therefore, we propose and test the notion that metabolic fingerprinting in a longitudinal study may characterize potential clinical biomarkers and provide new insights into the response to a particular treatment according to different BC phenotypes.

## 2 Materials and methods

### 2.1 Participants and ethics

A total of 92 BC female patients were enrolled in our study at the Medical Oncology Unit of the University Hospital of Jaén (Spain), in order to detect metabolomics changes associated with the efficiency of NACT. BC was divided into different subtypes by immunohistochemical and gene expression testing of the human epidermal growth factor 2 (HER2), hormone receptors of estrogen (ER) and progesterone (PR) and Ki‐67. Specifically, luminal B (LB) patients were diagnosed with HER2 negative (HER2−) and ER+ with a positive Ki‐67 finding defined as >15%. Patients who neither expressed hormone receptors (PR−, ER−) nor overexpressed HER2 were considered as triple‐negative (TN) patients; and, finally, patients overexpressing human epidermal growth factor 2 were diagnosed as HER2‐positive (HER2+) patients. Concretely, the evaluation of HER2 was done following the ASCO/CAP 2018 guidelines, by immunohistochemistry (IHC) staining and by fluorescent in situ hybridization (FISH): scores 0 and 1+ were considered negative, 3+ was considered HER2+, while a dual‐probe FISH was carried out for 2+ scores of the same specimen, or additional IHC or FISH for a new specimen [33]. Cancer stage was classified according to the 2010 Tumor Nodes Metastasis (TNM) system [34]. The main characteristics of these subjects are summarized in Table 1.

**Table 1:** Pathological and clinical characteristics of the subjects of study. N, nodes; P.R, pathologic response; post, postmenopause; pre, premenopause; T, tumor.

BC molecular subtypes	LB		TN		HER2+	
Subjects	48		21		23	
P.R	R	NR	R	NR	R	NR
	25	23	13	8	16	7
MP grading system						
MP1	—	1	—	2	—	1
MP2	—	3	—	2	—	1
MP3	—	19	—	4	—	5
MP4	14	—	5	—	5	—
MP5	11	—	8	—	11	—
Age (range)	49 (33–62)	52 (34–76)	53 (31–76)	48 (33–58)	48 (35–63)	58 (34–70)
BMI (Kg·m^−2^)	26 (19.3–38.7)	27 (20.1–36.5)	30 (22.1–41.7)	32 (22.1–38.9)	28 (19.6–40.6)	26 (19.0–32.5)
Menopausal status						
Pre	16	12	7	6	10	2
Post	9	11	6	2	6	5
HER2+ status	Negative		Negative		Positive	
PR Status	Neg/Pos		Negative		Neg/Pos	
ER Status	Positive		Negative		Neg/Pos	
Ki‐67	>15%		—		—	
Stage						
T1	5	2	0	0	0	0
T2	18	16	14	5	14	5
T3–4	2	5	2	2	2	2
N+	10	10	8	3	8	3
N−	14	13	6	4	6	4

Evaluation of potential confounding variables was performed using the Shapiro–Wilk normality test and, subsequently, Levene’s test for the equality of variances between responders (R) and nonresponders (NR), depending on age and body mass index (BMI) for each BC phenotype. U‐Mann and Whitney Wilcoxon test was performed for the data that presented a nonparametric distribution. The association analysis of the menopausal status with treatment response was checked with the Pearson chi‐square test. In addition, this statistical test allowed to evaluate whether the overall survival was related to the outcome to NACT in the TN phenotype. To know the intensity of the association, the Cramer’s V test was used. Venous blood samples were collected under fasting conditions at three different time points: before the first therapy cure with anthracyclines (basal); once they received taxol (presurgery); and after they went into surgery (postsurgery). The blood collection campaign was conducted over a timeframe period of eight years. Every patient provided a signed informed consent for participation prior to basal sample extraction. This study was approved by the institutional review board of the Clinical Research Ethics Committee of Jaén. All clinical investigations were conducted under Helsinki Declaration guidelines and International Conference on Harmonization‐Good Clinical Practices (ICH‐GCP).

### 2.2 Neoadjuvant chemotherapy

All patients received NACT consisting on bi‐weekly dose‐dense cycles of anthracyclines (epirubicin 90 mg·m^−2^ and cyclophosphamide 600 mg·m^−2^) followed by 12 weekly cycles of taxanes (paclitaxel 80 mg·m^−2^). Cycle time administration could be modified according to the Common Toxicity Criteria (CTC v5.0). Anti‐HER2 therapy (trastuzumab and pertuzumab) was added in HER2‐positive BC patients [35].

### 2.3 Response evaluation

Samples obtained during surgery underwent a histopathological analysis in order to determine the postsurgery Miller and Payne (MP) grade [36]. Pathological complete response was assessed from the five‐step scale based on reduction in malignant cellularity after treatment. Following these criteria, MP5 is considered as pCR with no malignant cells; MP4 is a very good response with <10% of malignant cells remaining, near the pCR; in MP3 the significant loss of tumor cells is too variable between 30 and 90%; MP2 shows a reduction of tumor cells < 30%, and MP1 has no reduction in malignant cells. Herein, we defined a response group (MP grades 4–5) and a nonresponse group (MP grades 1–3) according to the prognostic potential of the MP grading system [37, 38, 39, 40].

### 2.4 Sample collection and preparation

Blood samples were extracted using standard venipuncture processes and collected in EDTA tubes. Plasma was obtained by centrifugation at 1400× ***g*** for 10 min at 4 °C. All samples were kept at −80 °C until the analysis was made.

### 2.5 Metabolite extraction

An aliquot of 75 μL of plasma was mixed with 600 μL of cold acetonitrile (AcN) containing the analytical standard (roxithromycin). Then, it was shacked for 2 min at 2500 r.p.m. All the samples were centrifugated at 21 982 ***g*** for 10 min at 4 °C. Collected supernatants were transferred into new vials for evaporation and reconstituted in 210 μL of water/acetonitrile (50/50) with 0.1% formic acid.

### 2.6 Liquid chromatography coupled to high‐resolution mass spectrometry analysis

The analytical separation was achieved using liquid chromatography (LC) with an Agilent series 1290 (Agilent Technologies, Santa Clara, CA, USA) in reverse phase mode (RP) using Atlantis T3 C18 column (2.1 mm × 150 mm, 3 μm) from Waters (Water Corporation, Milford, MA, USA). The mobile phase A consisted of water/acetonitrile (90/10) and 0.1% formic acid. The mobile phase B consisted of acetonitrile/water (90/10) and 0.1% formic acid. The chromatographic run was 20 min. The gradient elution consisted of 0.0–0.5 min 1% eluent B; 0.5–11.0 min 99% eluent B, 11.0–15.5 min 99% eluent B and 15.5–15.6 min 1% eluent B. and 15.6–20.0 min 1% eluent B. Mass detection was performed using Triple TOF 5600 quadrupole time‐of‐flight mass spectrometer (SCIEX, Concord, ON, Canada). The mass spectrometer was operated using electrospray ionization in positive mode and an information‐dependent acquisition (IDA) method, and the eight most intense signals were fragmented. The exact mass calibration was automatically performed every six injections. Three different LC‐HRMS analyses were made in positive ionization mode, in order to detect molecular differences within the subtypes of BC (LB, TN, and HER2+) depending on their response to neoadjuvant chemotherapy after surgery. A total of 144 samples were analyzed for LB phenotype, 69 samples for HER2, and 63 for TN, and blanks and quality control (QC) samples were also used in each metabolomics analysis.

### 2.7 Data set creation

Peak View software (version 1.1.2; AB SCIEX) was used to evaluate the retention time and mass‐to‐charge (*m/z*) variability of three peaks over at different time points and *m/z* values. This allowed us to determine the ranges for the alignment. Peak detection, alignment, and data filtering were achieved using Marker view software (version 1.2.1; SCIEX). Collection parameters were set as follows: retention time window 0.10 min, noise threshold 70 cps, and mass tolerance 5.0 ppm. Additionally, only monoisotopic peaks were considered in order to decrease mass redundancy and improve true molecular features selection. Blank samples were used to remove contaminants and signals provided by solvents.

### 2.8 Analytical method validation and normalization

Principal component analysis (PCA) was used to assess the quality of the analytical system performance. QC samples clustering representation in this multivariate analysis (MVA) were useful to validate the analytical system's stability. The relative standard deviation (RSD) was calculated for all the features in the QC samples after the data set creation. Variables with variability higher than 30% were discarded (Table S1). Data normalization by a QC reference sample (probabilistic quotient normalization), logarithmic transformation, and autoscaling were performed in order to obtain a Gaussian‐type distribution.

### 2.9 Univariate statistical analysis

Two different statistical approaches were used in this work in order to determine the broadest range of metabolites that might differ between the groups of study when comparing them at a specific point or over time. Univariate statistical analysis was performed using the Student’s *t*‐test, which enabled assessing differences between R and NR patients of the TN, LB, and HER2+ molecular subtypes. Univariate statistical analysis (UVA) was applied at three different time points independently: before the first therapy cure with anthracyclines (basal, time 1), once the patients received treatment with taxol (presurgery, time 2), and after the breast‐conserving surgery (postsurgery, time 3). A *P‐*value < 0.05 was determined as the cutoff threshold with a Benjamini–Hochberg False Discovery Rate post hoc correction (FDR < 0.1). This analysis was carried out using Metaboanalyst 4.0 [41]. Eventually, discriminant metabolites selection was also based on their fold change (FC > 1.3).

### 2.10 Multivariate statistical analysis (ASCA)

The metabolomics data set shows a multilevel structure with multiple types of variation: the metabolic dynamism within the individual, the statistical differences between the subjects, and their combination. To deal with such complex information, we used ASCA, which factorizes the original data set into subsets describing the variation between response and nonresponse, the variation in time and their interaction [29]. To deal with unbalanced data, we used the ASCA+ version [42]. We tested for significance using exact and approximated permutation tests for the main factors (response, time, and patient) and interactions, respectively [43]. Significant factorized data were visualized using PCA. From statistically significant factors, we derived a list of relevant metabolites, ordered by the sum of squares of the difference between R and NR. All computations were done with the MEDA Toolbox for Matlab [44].

### 2.11 Identification of differential metabolites

Peak View software was used to establish a molecular formula according to the experimental exact mass, fragmentation spectrum, and isotope pattern. The identification of molecular components was achieved through comparative searches of available mass spectra using several databases such as Metlin, the Human Metabolome DataBase, Lipid Maps, NIST 2012, and mass bank mainly. Additional MS/MS analysis was carried out when necessary. Also, we used the information at the experimental conditions, ionization behavior, and/or retention time in order to assign a tentative identification. In those cases in which it was not possible to assign it, scientific literature was consulted. Finally, mass error of all the candidates was equal or lower than 5 ppm.

### 2.12 Biomarker evaluation

The area under the receiver‐operating characteristic curves was used to test the clinical relevance of candidate metabolites with corrected *P*‐value < 0.05. Assessment of the classifier performance was carried out with linear kernel support vector machine (SVM‐linear) and random forest models, using the Biomarker Analysis provided by Metaboanalyst.

## 3 Results

### 3.1 Patient’s characteristics

Regarding the patients eligible for analysis, a number of 55 BC subjects out of 92 were classified as R to NACT, in contrast to 37 NR subjects. Considering the BC phenotype, 16 out of 23 human epidermal growth factor 2 positive (HER2+) patients responded (69.56%) and 7 out of 23 showed a nonresponse according to the MP grading system (30.44%). In the case of luminal B (LB) molecular subtype, 26 out of 48 responded (54.16%), while 22 out of 48 did not show treatment response (45.84%). Last, the TN phenotype showed 13 out of 21 patients with response to NACT (61.9%) and 8 out of 21 patients with nonresponse (38.1%). Assessment of the confounding variables, body mass index, age, and menopausal status showed no significant differences in relation to response when the corresponding *t*‐test was applied for each BC phenotype (Table S2). In the case of the survival analysis, a moderate association with outcome to NACT was obtained for the TN phenotype (Table S3).

### 3.2 Metabolomic profiling from univariate analysis

Significant identified metabolites, selected according to *P*‐value corrected by FDR < 0.1 and FC > 1.3, are shown in Table 2. Other altered metabolites with *P*‐value < 0.05 (FDR > 0.1) and FC < 1.3 are identified in Table S4, and those not able to be identified are listed in Table S5. However, a lot of spectral information, and the availability of analytical standards, is still needed. In this study, tentative identities were classified at level 2 as reported by the Schymansky classification [45], validated by their MS/MS spectra (Fig. S1) after several searches in diverse databases (Metlin, Human Metabolome Database, Lipid Maps, NIST 2012 mass spectral library, or mass bank). Specifically, in the TN molecular subtype, a total of four signals were selected as significant at time 1 (t1) and time 2 (t2) but none at time 3 (t3). Candidate metabolites identified as cis‐4,7,10,13,16,19‐Docosahexaenoic acid and LysoPE (18:1) were found at t1. At t2, 2 significant metabolites were tentatively identified as 2 bile acids (glycodeoxycholic and glycohyocholic acid). The analysis for the LB phenotype showed three significant signals at t1 but none at t2 or t3. Candidate metabolites at t1 were tentatively identified as LysoPE (18:2), LysoPC (16:0), and tridecanoyl carnitine (Table 2).

**Table 2:** Tentative identification of the significant metabolites detected in the comparison between response groups in UVA. Δppm, mass error; FC, fold change > 1 indicates that the average normalized peak area ratio in R patients is larger than that in NR patients; RT, retention time; t1, before starting the therapy cure, basal level; t2, once the patients received taxol, presurgery; UVA, univariate analysis (Student’s *t‐*test).

Time point	BC molecular subtype	*m/z*	RT (min)	Molecular formula	Tentative identification	Δppm	Adduct	*P*‐value (FDR)	FC
t1	TN	329.246	14.39	C22H32O2	cis‐4,7,10,13,16,19‐Docosahexaenoic acid	0.3	[M + H]	0.059	2.198
		502.287	11.59	C23H46NO7P	LysoPE(18:1/0:0)	3.2	[M + Na]	0.059	−1.351
t1	LB	358.295	8.11	C20H39NO4	Tridecanoyl carnitine	1.2	[M + H]	0.032	−1.742
		478.293	10.79	C23H44NO7P	LysoPE(18:2/0:0)	0.4	[M + H]	0.084	1.352
		518.323	10.17	C24H50NO7P	LysoPC(16:0/0:0)	0.2	[M + Na]	0.032	1.694
t2	TN	448.305	8.45	C26H43NO6	Glycohyocholic acid	−1.5	[M + H − H_2_O]	0.004	3.404
		450.320	9.19	C26H43NO5	Glycodeoxycholic acid	0.7	[M + H]	0.004	3.967

Signals shown in Table S4, corresponding to 23 different *m/z* in TN, 2 in LB, and 1 in HER2+, would be expected to have significant values in larger and balanced cohorts. At the three time points, some *m/z* were detected as the same tentative identification with different adducts. There were 12 signals that could not be identified for the TN molecular subtype, 2 *m/z* for the LB, and no altered signals were detected at basal or at postsurgery levels, when comparing the response in HER2+ patients, as shown in Table S5.

### 3.3 Metabolic profile from multivariate analysis

**Fig. 1:**
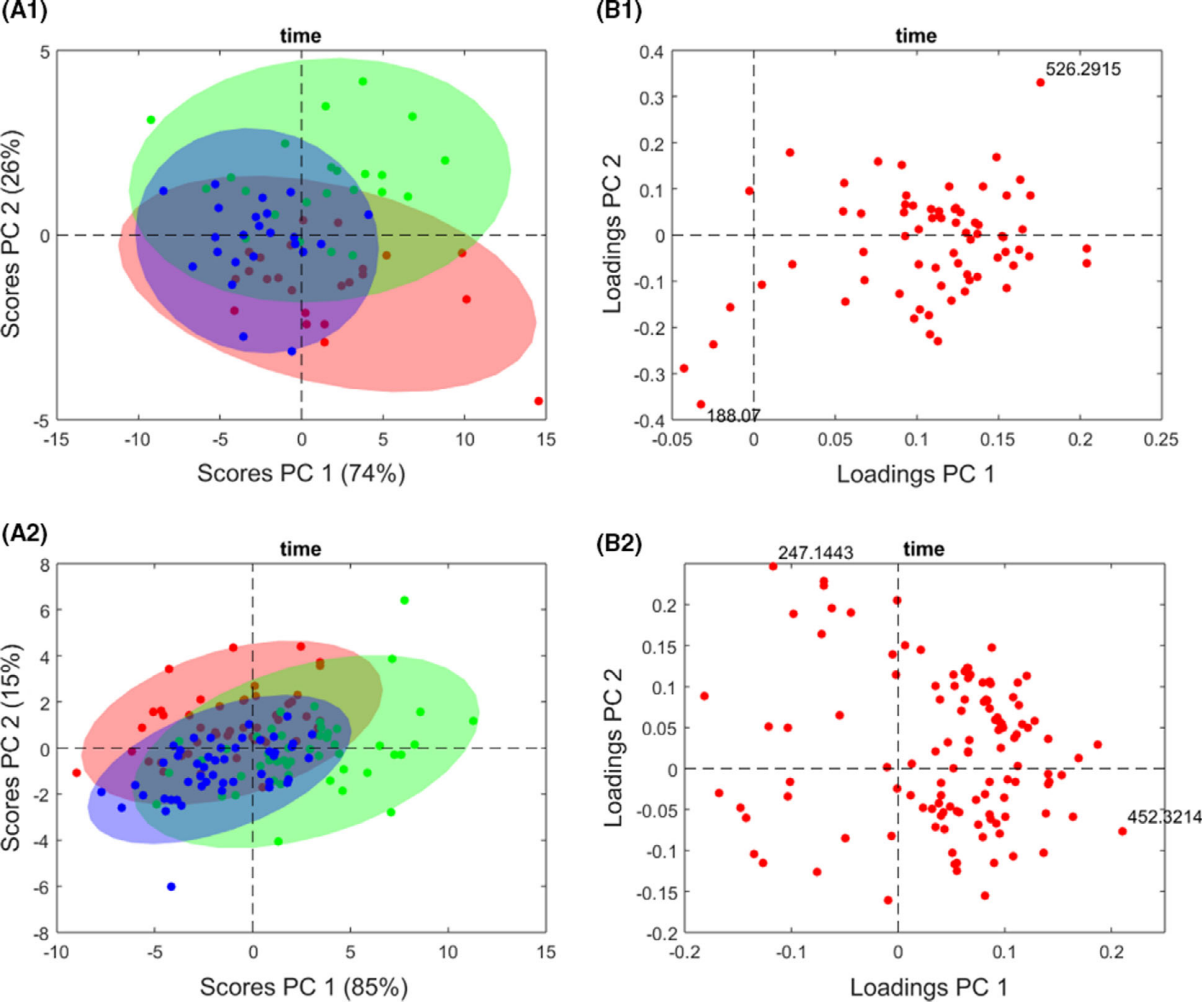
HER2+ and Luminal B phenotype longitudinal study using ANOVA–simultaneous component analysis (ASCA). The score plots represent the variation of the patient samples over time (basal, presurgery and postsurgery) in relation to the concentration of metabolites present in each of them, and the loading plots show the metabolites that are contributing to the significant differences over time in patients with luminal B and HER2+ phenotypes. (A1) 2D score plot of HER2+ patient samples over time. (A2) 2D score plot of Luminal B patient samples over time. (B1) The molecular ion at *m/z* 526.2915 [LysoPE (22:6/0:0)] and 188.07 (tryptophan) represent the metabolites most differential over time for the HER2+ phenotype. (B2) The molecular ion at *m/z* 247.1443 (tryptophan betaine) and 452.3214 represent the metabolites most differential over time for the luminal B phenotype. The red, blue, and green dots correspond to the basal, presurgery, and postsurgery time, respectively. [Colour figure can be viewed at wileyonlinelibrary.com]

ANOVA–simultaneous component analysis (ASCA) provided the statistically significant factors (Table 3) from which we drew up a list of associated relevant metabolites (Table 4). In our multivariate analysis, time and patient factors were statistically significant for the HER2+ and LB molecular subtypes (Figs S2 and S3), while response and patient factors were statistically significant for the TN (Fig. S4). To interpret the time factor, we used ASCA score and loading plots, that is, the PCA plots of the data factorized by ASCA. This is shown in Fig. 1 (A and B, respectively). Score plots in Fig. 1 illustrate samples of HER2+ (A1) and LB (A2) subjects corresponding to different time points (t1 in red, t2 in blue, and t3 in green), which can be interpreted in combination with the loading plots in Fig. 1B1,B2), where only most relevant signals are labeled (see also Table 4 and Table S6). Score plots include data ellipses at 0.05 significance level, although we did not use confidence levels, due to unbalanced data [46].

**Table 3:** Significant factors detected in ASCA.

BC molecular subtype	Factor	*P*‐value
TN	Patient Response	0.0020 0.0310
HER2+	Patient Time	0.001 0.001
LB	Patient Time	0.013 0.002

**Table 4:** Tentative identification of the metabolites significatively detected in ASCA. Δppm, mass error; RT, retention time.

BC molecular subtype	*m/z*	RT (min)	Molecular formula	Tentative identification	Δppm	Adduct
TN	448.3047^a^	8.45	C26H43NO6	Glycohyocholic acid	−1.5	[M + H − H_2_O]
	450.3200^a^	9.19	C26H43NO5	Glycodeoxycholic acid	0.7	[M + H]
	572.3699	11.87	C30H54NO7P	LysoPC (22:4/0:0)	0.6	[M + H]
HER2+	188.0700	3.57	C11H12N2O2	Tryptophan	0.5	[M + H − NH_3_]
	454.2922	11.19	C21H44NO7P	LysoPE (16:0/0:0)	−0.9	[M + H]
	566.3175	10.54	C28H50NO7P	LysoPC (20:4/0:0)	−1.3	[M + Na]
	583.2567	8.39	C33H34N4O6	Biliverdin	−0.9	[M + H]
	526.2915	10.62	C27H44NO7P	LysoPE (22:6/0:0)	−1.7	[M + H]
	568.3416	10.68	C30H50NO7P	LysoPC (22:6/0:0)	−2.2	[M + H]
	590.322	10.69	C30H50NO7P		−2.7	[M + Na]
LB	247.1443	3.86	C14H18N2O2	Tryptophan betaine	0.8	[M + H]
	342.2631	7.38	C19H35NO4	Dodecenoylcarnitine	−0.5	[M + H]
	363.2163	6.96	C21H30O5	Cortisol	0	[M + H]
	454.2935	11.36	C21H44NO7P	LysoPE (16:0/0:0)	0.2	[M + H]
	502.2921	10.5	C25H44NO7P	LysoPE (20:4/0:0)	−2	[M + H]

^a^ *m/z* found also as significant in univariate analysis.

For instance, the metabolite 526.2915 [LysoPE (22:6/0:0)] at the upper right corner of Fig. 1 (B1) is correlated with the green scores in Fig. 1 (A1), which represent HER2+ postsurgery measurements. Also, metabolite 188.07 (tryptophan) is right in the opposite direction. These signals can be identified as the ones that change the most after surgery (Fig. S5): 526.2915 and 188.07 present a generalized higher and lower value, respectively, after surgery. The same can be inferred in Fig. 1 (A2 and B2) but for 247.1443 (tryptophan betaine) and *m/z* 452.3214, with lower and higher values, respectively, after surgery in LB patients (Fig. S6).

**Fig. 2:**
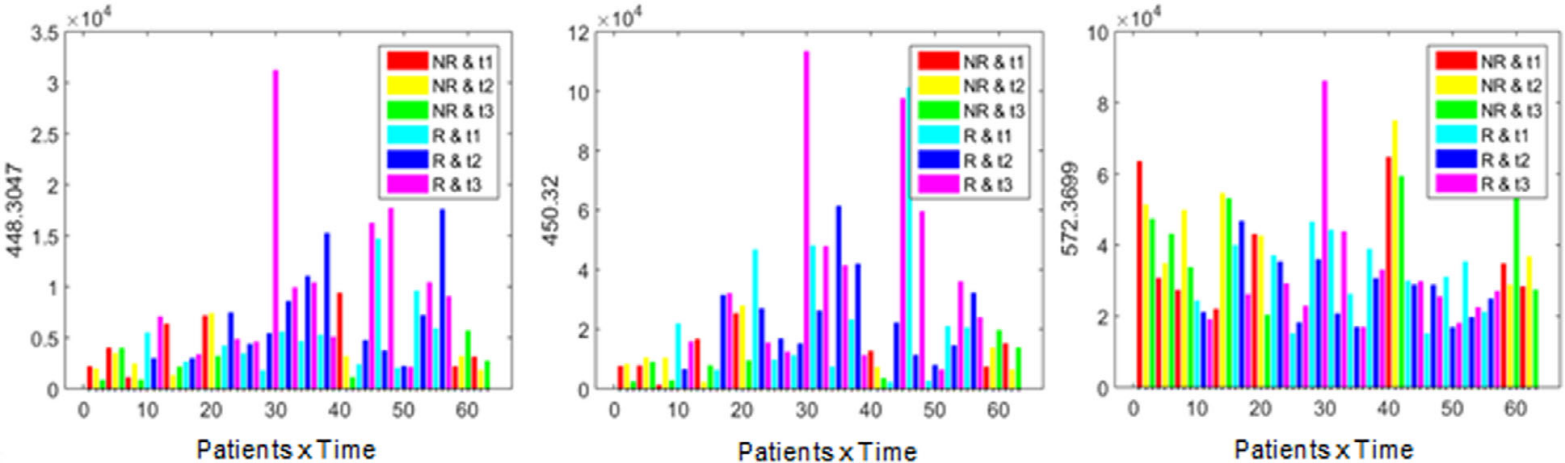
Differential metabolites according to the pathological response to neoadjuvant chemotherapy in triple‐negative breast cancer using phenotype ANOVA–simultaneous components analysis (ASCA). The molecular ions at *m/z* 448.3047 (glycohyocholic acid) and 450.32 (glycodeoxycholic acid) were found elevated in responders. The molecular ion at *m/z* 572.3699 [LysoPC (22:4)] appeared decreased in responders. R, responders; NR, nonresponders; t1, basal time; t2, presurgery; t3, postsurgery time. [Colour figure can be viewed at wileyonlinelibrary.com]

Lastly, ASCA of TN showed significance in response factor. Following the same approach using one PCA score/loading plots, we selected metabolites 448.3047 (glycohyocholic acid), 450.32 (glycodeoxycholic acid), and 572.3699 [LysoPC (22:4)] as the most differential between R and NR (Fig. 2). Metabolites 448.3047 and 450.32 in R tend to be generally higher than in NR, observation that agrees with significant results after FDR correction in Table 2. Otherwise, *m/z* 572.3699 tends to be mostly higher in NR than in R.

### 3.4 Candidate biomarker evaluation

Significant metabolites were checked for their diagnostic potential with a multivariate receiver‐operating characteristic (ROC) analysis. The area under curve (AUC) obtained for the 448.3047 (glycohyocholic acid) and 450.32 (glycodeoxycholic acid) in combination (0.946, 95% CI: 0.875–1) indicates how well these candidate biomarkers distinguish between our groups of study (Fig. S7a). Based on this model, only 3 out of 13 TN R were wrongly classified as NR, whereas all TN NR were correctly classified (Fig. S7b). Finally, the prognostic power of these bile acids in combination was tested with an AUC performance of 0.777 (95% CI: 0.541–1). The model indicates a good classification of patient subgroups with survival expectancy of more than 2 years (Fig. S8). However, an independent cohort would be required to validate the prognostic power of these promising candidates.

## 4 Discussion

Neoadjuvant chemotherapy constitutes a standard treatment for the management of BC with several benefits, although there are yet unresolved questions that concern a high percentage of women that suffer from this heterogeneous disease. Some challenges faced in the clinical practice that affect the efficiency of this systemic treatment are the lack of early predictors of response, as well as the establishment of the pCR prognostic value. Stratification of BC patients according to underlying molecular factors that confer NACT resistance would be a great step toward personalized medicine.

In this work, the untargeted LC‐HRMS‐based metabolomics approach used enables the detection of different small molecules that may be involved in the behavior of three BC phenotypes against NACT. For this purpose, two statistical analyses—univariate and multivariate—were carried out. As an outcome of UVA, alteration of the metabolome in LB and HER2+ subjects only appeared at basal or presurgery levels, while the TN molecular subtype showed the highest variability in response to treatment at all time points. The use of ASCA is of great relevance for better understanding the greater metabolome impact over time and to properly select the biomarkers that might be potential predictors of the chemotherapy response associated with the phenotype. This prominent multivariate method allows analyzing complex metabolomics data sets with simultaneously measured covariates considering the experimental design [26, 29, 31, 32, 47].

Thus, our longitudinal study analyzed the influence that factors such as the individual itself, response to treatment, time, and their interaction, may have on the dynamic metabolome of 92 BC patients. Clearly, the significance of the patient factor, obtained in the ASCA results of all the molecular subtypes studied, reflects the need for a tailored follow‐up in BC [14, 16, 48, 49, 50]. On the contrary, the significance of the time factor (with nonsignificant response) should be interpreted as a homogeneous change in the metabolome of the HER2+ and LB patients after treatment, regardless they are classified as R or NR.

Regarding the outcomes obtained in the LB and HER2+ analyses, we highlight the reprogramming of cellular metabolism as a hallmark of BC. Herein, in the UVA of LB molecular subtype, lysophospholipids were increased at basal levels of responder patients while carnitines appeared as decreased. So that, we suggest that phospholipids (PL) and carnitines may be considered as useful targets for cancer therapy and as BC biomarkers, as described in previous observations [51, 52, 53, 54, 55]. In addition, alteration of amino acids was also found. The lower tryptophan betaine levels detected postsurgery in LB patients could give insights into its potential role in the phenotype behavior [56, 57]. Likewise, research on larger cohorts would help to validate whether the increased concentration of LysoPE (22:6) at t3 in HER2+ could be a decisive biomarker of residual disease. It should be especially noted the dysregulation of the tryptophan (Trp) metabolism in the HER2+ molecular subtype. Specifically postsurgery, a significant decrease in Trp plasmatic concentrations was observed, as previously reported in serum and plasma of BC patients [58, 59, 60]. In this regard, Trp catabolism dysregulation is known to indirectly contribute to cancer progression by the kynurenine (Kyn) pathway [61, 62, 63], although no associations with response or sensitivity to chemotherapy were observed in previous studies, which coincides with our observations [22, 64]. In this line, further investigating the metabolome alteration related to treatment response is still needed to better understand the behavior of the LB and HER2+ molecular subtypes in response to NACT.

Unlike the molecular subtypes LB and HER2+, this approach notably differentiates TN patients that respond to NACT and those who do not. From the ASCA results, given that treatment response is statistically significant, but time is not, we could conclude that there is a difference between R and NR sustained across the three time points in the TN phenotype. In particular, this metabolic difference may relate to treatment effectiveness and, if validated in future analyses, to treatment selection. It should be pointed out that, while response over time was not found to be significant, the effect size of this interaction in TN doubles the one obtained in HER2+ and triples the one in LB. Hence, it may be of great interest to further investigate the interaction between time and response in order to determine the prognostic applicability of the candidate biomarkers proposed for treatment efficiency prediction in BC phenotypes.

In our findings, both statistical strategies supplement the results in the TN analyses in relation to response. At basal plasma levels, the docosahexaenoic acid (DHA) concentrations are significantly higher in TN R than in NR. The dysregulation of DHA is of great importance since it has been shown to be involved in cell signaling, leading to the reduction in cancer cell viability and proliferation both in vivo and in vitro [65, 66, 67]. Indeed, DHA supplementation in combination with NACT is being explored in the interventional study NCT03831178 (ClinicalTrial.gov). This observation clearly supports that the measurement of this fatty acid may be considered as a biomarker for an early detection of chemoresistance at the diagnosis of the disease.

Furthermore, two bile acids (BAs), glycodeoxycholic acid (GDCA) and glycohyocholic acid (GHCA), were significant in TN presurgery R. The predictive biomarker model with these candidates was evaluated with a multivariate ROC analysis, which showed excellent performance since all the TN NR were correctly classified at t2. Their prognostic power was also assessed, obtaining a good classification between patients with survival expectancy of more than 2 years. Additionally, the role of bile acids in carcinogenesis is increasingly being studied. Thus, paradoxical functions of these bioactive molecules have been observed depending on the tissue affected and BA receptor activation (FXRa, TGR5) in cancer [68, 69]. However, not many studies have been able to shed light on how their dysregulation may affect BC development and behavior [70, 71, 72]. Nonetheless, we observed that plasma levels of conjugated secondary bile acids, GDCA and GHCA, are higher in TN R when compared to NR at pre‐ and postsurgery time points. Nevertheless, GDCA and GHCA were not found at basal levels, which may be the reason why interaction between response and time factors is not significant in our ASCA outcome. Being secondary BAs directly related to the intestinal microbiota, the study of its potential role in the behavior of BC should be investigated to a greater extent [73]. In this regard, different clinical trials gather more information about the effect of chemotherapy on gut bacteria and the affection that gut microbiota might have on the NACT‐induced immunosurveillance in TN patients (NCT02370277 and NCT03586297, ClinicalTrial.gov). Notwithstanding these promising results, further analysis would be needed in order to better understand the effects of medical interventions on the microbiome, as well as the relevance of independent bile acids as constituents of the BC tumor microenvironment. Thus, a good noninvasive prognostic strategy for the aggressive TN phenotype is suggested in this study by detection of BAs in plasma using LC‐HRMS.

On the contrary, variations in the composition of plasma phospholipids compared with the treatment response appeared at different time points in our analyses. Specifically, the increased concentration of phosphatidylethanolamines [LysoPE (18:1) and (18:2)] at t1 in TN NR is supported by the increased demand for PE in BC cells under metabolic stress [74, 75]. Otherwise, the phosphatidylcholine LysoPC (22:4) was determined from the ASCA results as a significantly increased metabolite for nonresponder TN patients. In line with our findings, it could be inferred that evolving knowledge of these candidate metabolites’ behavior in the BC process would improve the stratification of the BC patients for better therapy decision‐making.

## 5 Conclusion

In conclusion, our work presents dynamic metabolic changes at the individual level in all the phenotypic analyzes carried out during disease and treatment. The complete set of small molecules within a biological sample can be influenced by pathological processes, treatment, as well as the microbiome, thus affecting its consequent relationship with the metabolome. The high level of individual variability makes it difficult to find a single metabolic signature to classify our groups of patients. Nevertheless, the results obtained in TN subtype between R and NR may point toward new approaches in the fight against cancer. A larger sample size and number of balanced cohorts would help to corroborate and validate the findings reported in this work. Lastly, the combination of untargeted metabolomics and ASCA appears to be a highly valuable tool for deciphering the behavior of BC treated with NACT and, thus, open up the possibility of an early modification of this therapy according to the future response to treatment, improving prognosis for these patients.

## Conflict of interest

The authors declare no conflict of interest.

## Author contributions

CD, CG‐O, and LD‐B contributed to conceptualization, methodology, formal analysis, investigation, resources, data curation, writing—original draft preparation, writing—review and editing, and visualization. JC contributed to methodology, software, formal analysis, data curation, writing—original draft preparation, and writing—review and editing. PMG contributed to methodology. AM‐B contributed to methodology. MF‐N contributed to investigation and resources. ALO‐G contributed to resources. FG‐M contributed to resources. JAM contributed to writing—review and editing and visualization. FV contributed to resources, writing—review and editing, and supervision. JPdP contributed to writing—review and editing and visualization. PS‐R contributed to conceptualization, writing—review and editing, visualization, project administration, and funding acquisition. All authors have read and agreed to the published version of the manuscript.

### Peer review

The peer review history for this article is available at https://publons.com/publon/10.1002/1878‐0261.13216.

## Supporting information


**Fig. S1.** Experimental MS/MS spectrum obtained in our analysis for the secondary bile acids a) glycodeoxycholic acid and b) glycohyocholic acid.
**Fig. S2.** Reference distribution for HER2+ significance testing with resampling in ANOVA–simultaneous component analysis: time factor (left, *P*‐value = 0.002) and patient factor (right, *P*‐value = 0.013).
**Fig. S3.** Reference distribution for LB significance testing with resampling in ANOVA–simultaneous component analysis: time factor (left, *P*‐value = 0.001) and patient factor (right, *P*‐value = 0.001).
**Fig. S4.** Reference distribution for TN significance testing with resampling in ANOVA–simultaneous component analysis: time factor (left, *P*‐value = 0.031) and patient factor (right, *P*‐value = 0.002).
**Fig. S5.** Differential expression of 526.2915 [LysoPE (22:6) and 188.07 (tryptophan)] according to the pathological response group (R, responders; NR, nonresponders) in HER2+ at time 1 (t1, basal), time 2 (t2, presurgery) and time 3 (t3, postsurgery) detected using ANOVA–simultaneous component analysis.
**Fig. S6.** Differential expression of 247.1443 (tryptophan betaine) and 452.3214 (not identified) according to the pathological response group (R, responders; NR, nonresponders) in LB at time 1 (t1, basal level), time 2 (t2, presurgery), and time 3 (t3, postsurgery) detected using ANOVA–simultaneous component analysis.
**Fig. S7.** ROC curve plot for the model obtained from combination of the significant candidates identified in TN breast cancer molecular subtype [448.3047 (glycohyocholic acid) and 450.32 (glycodeoxycholic acid)]: (a) ROC curve plot was created from the averaged results of 100 cross‐validations; (b) as an outcome the model provides with the distinction of all nonresponders TN patients and 3 out of 13 responders misclassified.
**Fig. S8.** ROC curve plot for the prognostic model obtained from combination of the significant candidates identified in TN breast cancer molecular subtype [448.3047 (glycohyocholic acid) and 450.32 (glycodeoxycholic acid)]: (a) ROC curve plot was created from the averaged results of 100 cross‐validations; (b) as an outcome the model provides with the distinction of 2 out of 7 patients from the nonsurvival group and 5 out of 14 survivors misclassified.
**Table S1.** Selected variables from the untargeted metabolomics analysis for each breast cancer molecular subtype.
**Table S2.** Values of significance for normality and homoscedasticity tests of the continuous variables: age and BMI; and for association tests of the categorical variable: menopausal status.
**Table S3.** Association tests of the survival and treatment response data in the TN phenotype.
**Table S4.** Tentative identification of the differential metabolites between response groups in UVA.
**Table S5.** Differential signals between response groups without a tentative identification according to the breast cancer molecular subtype detected in UVA.
**Table S6.** Differential signals without a tentative identification detected in ASCA according to time and patient factors.

## Data Availability

The data analyzed and generated in our work are available upon request from the corresponding author. The data are not publicly available due to patient confidentiality, participant privacy, and ethical restrictions.
