# Supplementary material for: Predicting dynamic response to neoadjuvant chemotherapy in breast cancer: a novel metabolomics approach
Source: Mol Oncol. 2022 Apr 14;16(14):2658–71. doi: 10.1002/1878-0261.13216 (PMC9297806; doi:10.1002/1878-0261.13216)
Supplement: Supplementary file 1 — Fig. S1. Experimental MS/MS spectrum obtained in our analysis for the secondary bile acids a) glycodeoxycholic acid and b) glycohyocholic acid. Fig. S2. Reference distribution for HER2+ significance testing with resampling in ANOVA–simultaneous component analysis: time factor (left, P‐value = 0.002) and patient factor (right, P‐value = 0.013). Fig. S3. Reference distribution for LB significance testing with resampling in ANOVA–simultaneous component analysis: time factor (left, P‐value = 0.001) and patient factor (right, P‐value = 0.001). Fig. S4. Reference distribution for TN significance testing with resampling in ANOVA–simultaneous component analysis: time factor (left, P‐value = 0.031) and patient factor (right, P‐value = 0.002). Fig. S5. Differential expression of 526.2915 [LysoPE (22:6) and 188.07 (tryptophan)] according to the pathological response group (R, responders; NR, nonresponders) in HER2+ at time 1 (t1, basal), time 2 (t2, presurgery) and time 3 (t3, postsurgery) detected using ANOVA–simultaneous component analysis. Fig. S6. Differential expression of 247.1443 (tryptophan betaine) and 452.3214 (not identified) according to the pathological response group (R, responders; NR, nonresponders) in LB at time 1 (t1, basal level), time 2 (t2, presurgery), and time 3 (t3, postsurgery) detected using ANOVA–simultaneous component analysis. Fig. S7. ROC curve plot for the model obtained from combination of the significant candidates identified in TN breast cancer molecular subtype [448.3047 (glycohyocholic acid) and 450.32 (glycodeoxycholic acid)]: (a) ROC curve plot was created from the averaged results of 100 cross‐validations; (b) as an outcome the model provides with the distinction of all nonresponders TN patients and 3 out of 13 responders misclassified. Fig. S8. ROC curve plot for the prognostic model obtained from combination of the significant candidates identified in TN breast cancer molecular subtype [448.3047 (glycohyocholic acid) and 450.32 (glycode [file MOL2-16-2658-s001.docx]

| **Supplementary Table 1.** Selected variables from the untargeted metabolomics analysis for each breast cancer molecular subtype. | | | | | |
| --- | --- | --- | --- | --- | --- |
|  |  |  |  |  |  |
| **BC molecular subtype** | **Total** | **Monoisotopics** | **Contaminant’s filtering** | **Discarded (RSD>30%)** |  |
| **LB** | 2670 | 946 | 124 | 117 |  |
| **TN** | 2378 | 968 | 113 | 112 |  |
| **HER2+** | 2537 | 775 | 71 | 70 |  |

BC: breast cancer; LB: luminal B; TN: triple negative; HER2+: human epidermal growth factor receptor 2 positive; RSD: relative standard deviation

**Supplementary Table 2.** Values of significance for normality and homoscedasticity tests of the continuous variables: age and BMI; and for association tests of the categorical variable: menopausal status.

| **BC Molecular Subtype** | **Shapiro-Wilk** | **Levene's Test** | **U Mann Whitney** | **Pearson**  **Chi**  **Square** | **Cramer’s**  **V** |  |
| --- | --- | --- | --- | --- | --- | --- |
| **Age** | TN | 0.362 | 0.401 | NA | NA | NA |
|  | LB | 0.495 | 0.111 | NA | NA | NA |
|  | HER2+ | 0.424 | 0.124 | NA | NA | NA |
| **BMI** | TN | 0.159 | 0.989 | NA | NA | NA |
|  | LB | 0.016 | NA | 0.09 | NA | NA |
|  | HER2+ | 0.109 | 0.21 | NA | NA | NA |
| **Menopausal**  **Status** | TN | NA | NA | NA | 0.112 | NA |
|  | LB | NA | NA | NA | 0.281 | NA |
|  | HER2+ | NA | NA | NA | 0.134 | NA |

BC: breast cancer; TN: triple negative; LB: luminal B; HER2+: human epidermal growth factor receptor 2 positive; BMI: body mass index; NA: not applicable

**Supplementary Table 3.** Association tests of the survival and treatment response data in the TN phenotype.

| **BC Molecular Subtype** | TN | |  |
| --- | --- | --- | --- |
| **Subjects** |  | 21 | |
| **P.R** |  | **R** | **NR** |
|  |  | 13 | 8 |
| **Overall Survival** | > 24 months  exitus | 11  2 | 3  5 |
| **(median)** | follow up time, months | 53 | 39 |
| **Association tests** | **Pearson**  **Chi**  **Square** | 0.026 | |
|  | **Cramer’s**  **V** | 0.485 | |

BC: breast cancer**;** TN: triple negative; R: responders; NR: non-responders; P.R: pathologic response

**Supplementary Table 4.** Tentative identification of the differential metabolites between response groups in UVA.

| **Time point** | **BC molecular subtype** | **m/z** | **RT**  **(min)** | **Molecular formula** | **Tentative identification** | **Δppm** | **Adduct** | ***p –* value*** | **FC** |
| --- | --- | --- | --- | --- | --- | --- | --- | --- | --- |
| **t1** | **LB** | 450.321 | 7.76 | C26H43NO5 | Glycodeoxycholic acid | -0.2 | [M+H] | 0.045 | 1.625 |
|  |  | 478.293 | 10.79 | C23H44NO7P | LysoPE(18:2/0:0) | 0.4 | [M+H] | 0.002 | 1.352 |
| **t1** | **TN** | 480.306 | 11.76 | C23H46NO7P | LysoPE(18:1/0:0) | 0.3 | [M+H] | 0.009 | 1.37 |
|  |  | 1039.67 | 10.83 | C26H50NO7P | LysoPC(18:2/0:0) | 2 | [2M+H] | 0.022 | 1.529 |
|  |  | 478.294 | 10.64 | C23H44NO7P | LysoPE(18:2/0:0) | 0.4 | [M+H] | 0.021 | -1.340 |
|  |  | 500.274 | 10.62 | C23H44NO7P |  | -0.7 | [M+Na] | 0.012 | -1.365 |
|  |  | 508.341 | 11.01 | C25H50NO7P | LysoPC(17:1/0:0) | -0.5 | [M+H] | 0.0099 | 1.383 |
| **t2** |  | 414.301 | 8.94 | C26H43NO5 | Glycodeoxycholic acid | -0.2 | [M+H-2H2O] | 0.021 | 1.658 |
|  |  | 432.311 | 8.96 | C26H43NO5 |  | 0.4 | [M+H-H2O] | 0.019 | 1.706 |
|  | **TN** | 450.323 | 8.94 | C26H43NO5 |  | -0.4 | [M+H] | 0.024 | 1.671 |
|  |  | 472.303 | 8.94 | C26H43NO5 |  | -0.9 | [M+Na] | 0.012 | 1.744 |
|  |  | 466.317 | 7.62 | C26H43NO6 | Glycocholic acid | -2.4 | [M+H] | 0.006 | 2.183 |
|  |  | 496.338 | 11.27 | C24H50NO7P | LysoPC(O-14:0/2:0) | 0.1 | [M+H] | 0.015 | -1.339 |
|  |  | 991.672 | 11.29 | C48H100N2O14P2 |  | 0.1 | [2M+H] | 0.045 | -1.980 |
|  |  | 542.323 | 10.03 | C28H48NO7P | LysoPC(20:5/0:0) | 0 | [M+H] | 0.010 | 1.343 |
|  |  | 564.305 | 10.02 | C28H48NO7P |  | 0.2 | [M+Na] | 0.009 | 1.323 |
|  |  | 566.321 | 10.74 | C28H50NO7P | LysoPC(20:4/0:0) | 0.2 | [M+Na] | 0.029 | -1.437 |
| **t2** | **HER2+** | 426.357 | 9.89 | C25H47NO4 | Oleoylcarnitine | -3.3 | [M+H] | 0.045 | -1.365 |
| **t3** | **TN** | 414.301 | 8.94 | C26H43NO5 | Glycodeoxycholic acid | -0.2 | [M+H-2H2O] | 0.006 | 2.25 |
|  |  | 432.310 | 8.96 | C26H43NO5 |  | 0.4 | [M+H-H2O] | 0.005 | 2.44 |
|  |  | 450.32 | 9.19 | C26H43NO5 |  | 0.7 | [M+H] | 0.004 | 2.22 |
|  |  | 450.323 | 8.94 | C26H43NO5 |  | -0.4 | [M+H] | 0.008 | 2.5 |
|  |  | 472.303 | 8.94 | C26H43NO5 |  | -0.9 | [M+Na] | 0.005 | 2.3 |
|  |  | 448.305 | 8.45 | C26H43NO6 | Glycohyocholic acid | -1.5 | [M+H-H2O] | 0.001 | 2.31 |
|  |  | 466.317 | 7.62 | C26H43NO6 | Glycocholic acid | -2.4 | [M+H] | 0.034 | 1.71 |
|  |  | 524.374 | 12.77 | C26H54NO7P | LysoPC(O-16:0/2:0) | -1.3 | [M+H] | 0.028 | -1.471 |
|  |  | 572.370 | 11.87 | C30H54NO7P | LysoPC(22:4/0:0) | 0.6 | [M+H] | 0.008 | -1.754 |
|  |  | 991.672 | 11.29 | C48H100N2O14P2 | LysoPC(O-14:0/2:0) | 0.1 | [2M+H] | 0.018 | -1.667 |

UVA: univariate analysis (Student’s *t*-test); BC: breast cancer; TN: triple negative; LB: luminal B; HER2+: human epidermal growth factor receptor 2 positive; m/z: mass-to-charge ratio; RT: retention time; *p*–value*: *p*-value which FDR > 0.1; FC: fold change > 1 indicates that the average normalized peak area ratio in responder patients is larger than that in non-responder patients; t1: before starting the therapy cure at basal level; t2: once the patients received taxol, pre-surgery; t3: after going to the breast conserving surgery, post-surgery

**Supplementary Table 5.** Differential signals between response groups without a tentative identification according to the breast cancer molecular subtype detected in UVA.

| **Time point** | **BC molecular subtype** | **m/z** | **RT (min)** | ***p–*value*** | **FC** |
| --- | --- | --- | --- | --- | --- |
| **t1** | **LB** | 546.7947 | 10.83 | 0.028 | 1.515 |
|  |  | 1041.681 | 11.43 | 0.018 | 1.350 |
| **t2** | **TN** | 754.9921 | 11.29 | 0.021 | -1.567 |
|  |  | 755.9839 | 11.27 | 0.044 | -1.519 |
|  |  | 762.9844 | 11.27 | 0.045 | -1.516 |
|  |  | 765.9889 | 11.27 | 0.011 | -1.413 |
|  |  | 1010.65 | 11.29 | 0.044 | -1.980 |
|  |  | 1013.65 | 11.32 | 0.019 | -1.926 |
|  |  | 1258.309 | 11.29 | 0.032 | -1.442 |
| **t3** | **TN** | 754.9921 | 11.29 | 0.023 | -1.515 |
|  |  | 755.9839 | 11.27 | 0.049 | -1.515 |
|  |  | 1002.6574 | 11.29 | 0.018 | -1.786 |
|  |  | 1013.6496 | 11.32 | 0.023 | -1.449 |
|  |  | 1258.3088 | 11.29 | 0.018 | -2.128 |

UVA: univariate analysis (Student’s *t*-test); BC: breast cancer; TN: triple negative; LB: luminal B; m/z: mass-to-charge ratio; RT: retention time; *p* – value*: *p*-value which FDR > 0.1; FC: fold change > 1 indicates that the average normalized peak area ratio in responder patients is larger than that in non-responder patients; t1: basal level; t2: pre-surgery; t3: post-surgery

**Supplementary Table 6.** Differential signals without a tentative identification detected in ASCA according to time and patient factors.

| **BC molecular subtype** | **m/z** | **RT**  **(min)** |
| --- | --- | --- |
| **HER2+** | 576.3276 | 10.75 |
| **LB** | 452.3214 | 8.99 |
|  | 515.2623 | 3.86 |
|  | 409.1604 | 9.3 |

BC: breast cancer; HER2+: human epidermal growth factor receptor 2 positive; LB: luminal B; m/z: mass-to-charge ratio; RT: retention time

**
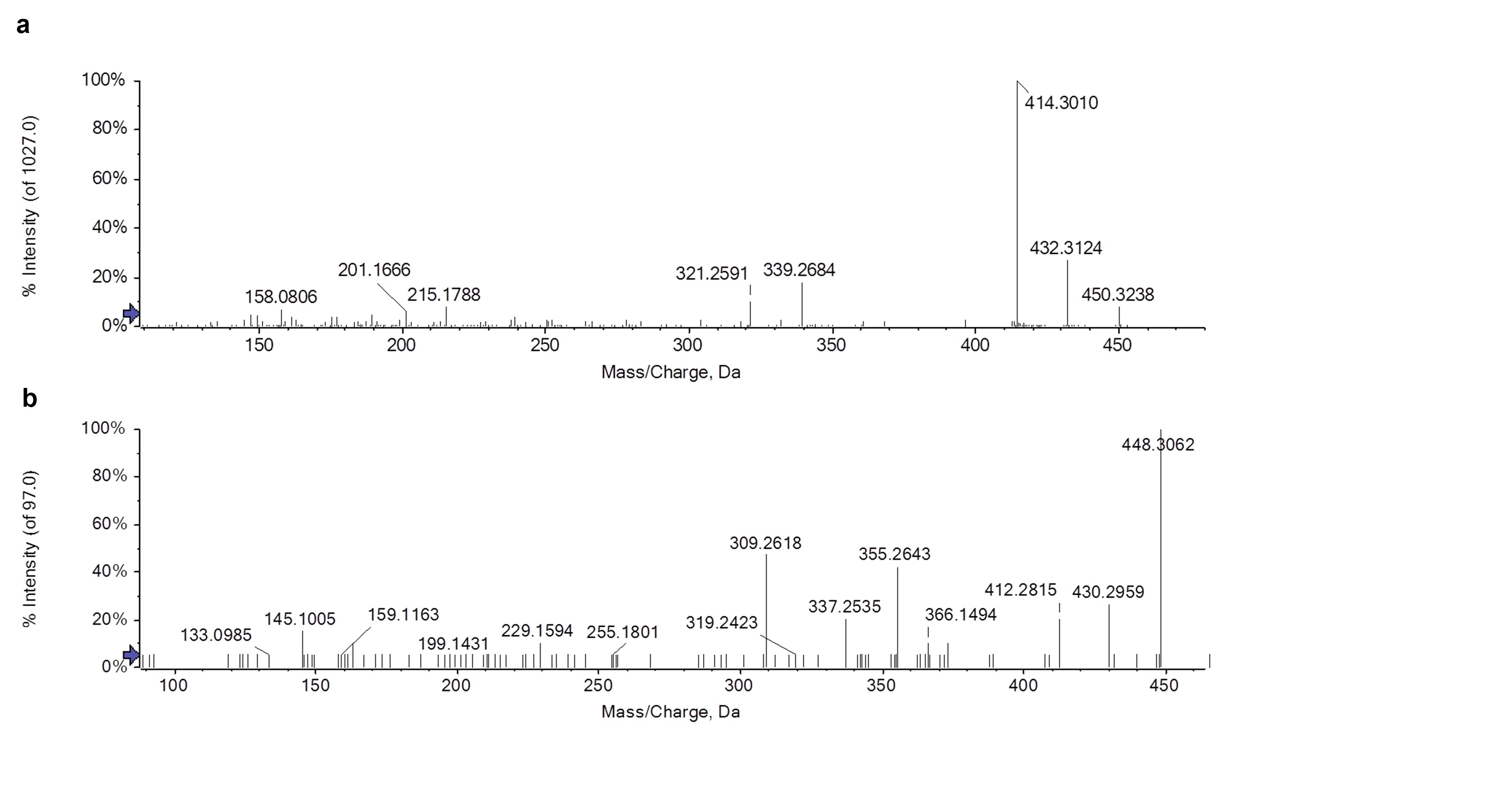
**

**Supplementary Figure 1** Experimental MS/MS spectrum obtained in our analysis for the secondary bile acids a) glycodeoxycholic acid and b) glycohyocholic acid.


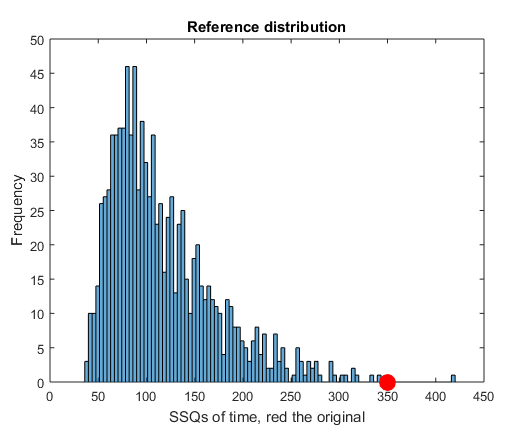

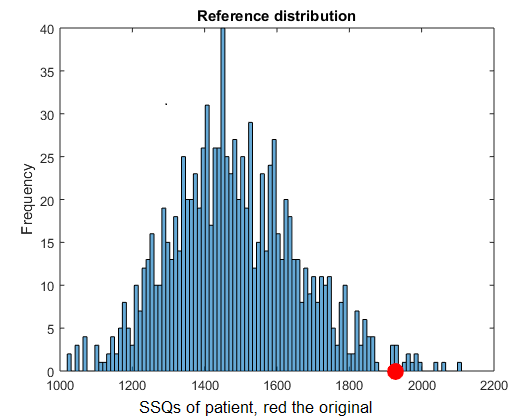


**Supplementary Figure 2**. Reference distribution for HER2+ significance testing with resampling in ANOVA-simultaneous component analysis: time factor (left, *p* – value = 0.002) and patient factor (right, *p* - value = 0.013).

| 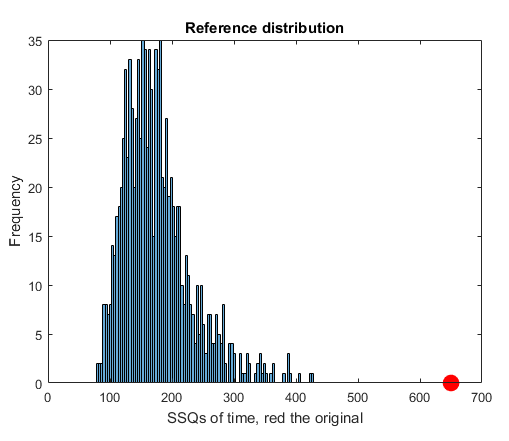 | 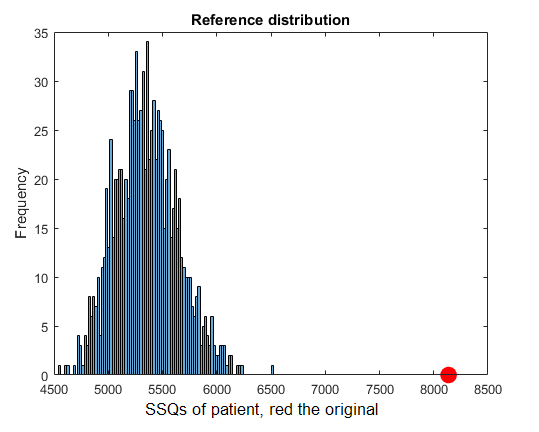 |
| --- | --- |

**Supplementary Figure 3**. Reference distribution for LB significance testing with resampling in ANOVA-simultaneous component analysis: time factor (left, *p*-value = 0.001) and patient factor (right, *p*-value = 0.001).

| 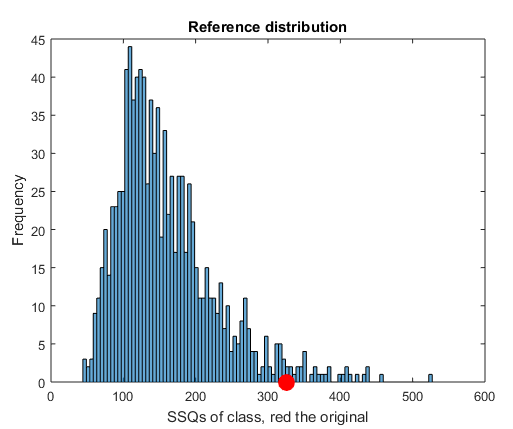 | 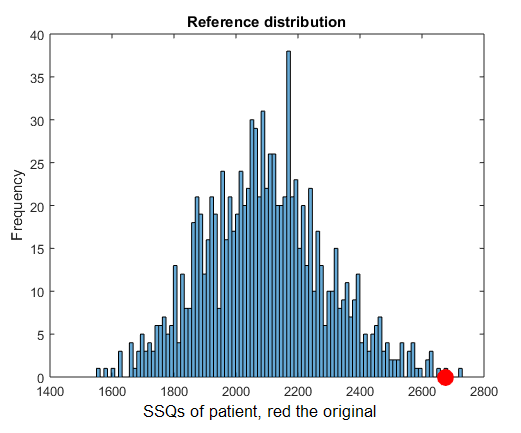 |
| --- | --- |

**Supplementary Figure 4**. Reference distribution for TN significance testing with resampling in ANOVA-simultaneous component analysis: time factor (left, *p*-value = 0.031) and patient factor (right, *p*-value = 0.002).


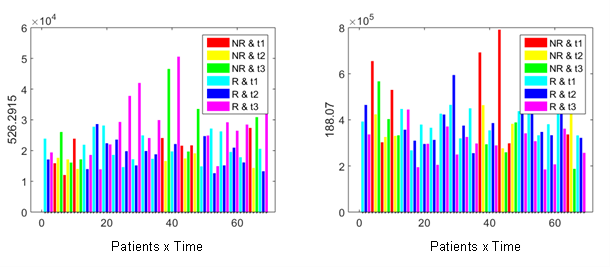


**Supplementary Figure 5.** Differential expression of 526.2915 (LysoPE (22:6) and 188.07 (tryptophan) according to the pathological response group (R, responders; NR, non-responders) in HER2+ at time 1 (t1, basal), time 2 (t2, pre-surgery) and time 3 (t3, post-surgery) detected using ANOVA-simultaneous component analysis.


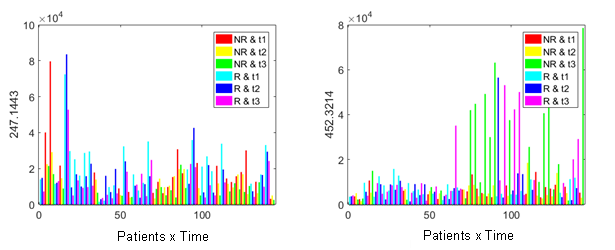


**Supplementary Figure 6**. Differential expression of 247.1443 (tryptophan-betaine) and 452.3214 (not identified) according to the pathological response group (R, responders; NR, non-responders) in LB at time 1 (t1, basal level), time 2 (t2, pre-surgery) and time 3 (t3, post-surgery) detected using ANOVA-simultaneous component analysis.

**
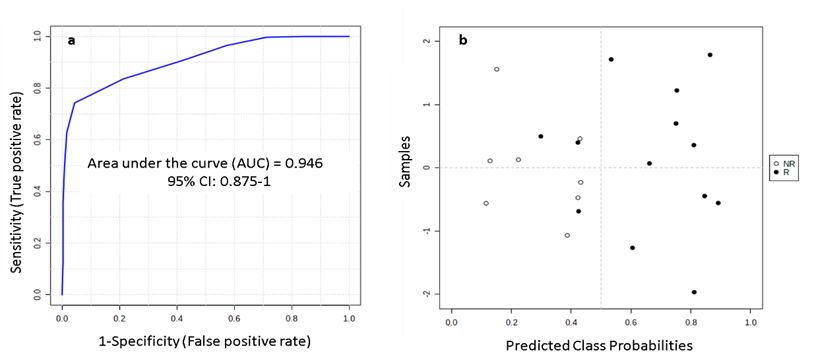
**

**Supplementary Figure 7**. ROC curve plot for the model obtained from combination of the significant candidates identified in TN breast cancer molecular subtype (448.3047 (glycohyocholic acid) and 450.32 (glycodeoxycholic acid)): (a) ROC curve plot was created from the averaged results of 100 cross-validations; (b) as an outcome the model provides with the distinction of all non-responders TN patients and 3 out of 13 responders misclassified.


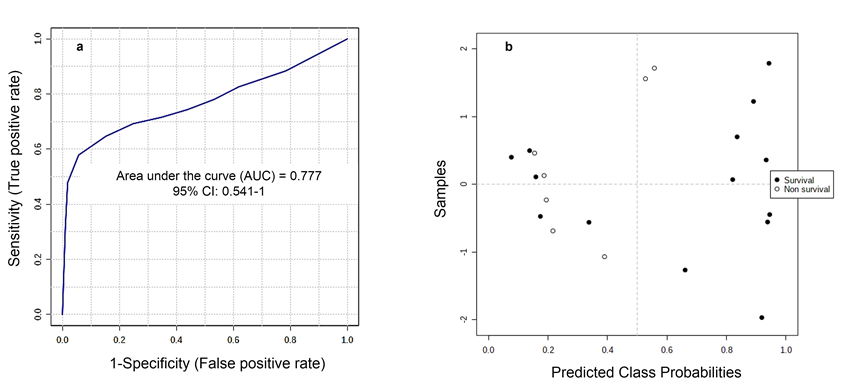


**Supplementary Figure 8**. ROC curve plot for the prognostic model obtained from combination of the significant candidates identified in TN breast cancer molecular subtype (448.3047 (glycohyocholic acid) and 450.32 (glycodeoxycholic acid)): (a) ROC curve plot was created from the averaged results of 100 cross-validations; (b) as an outcome the model provides with the distinction of 2 out of 7 patients from the non-survival group and 5 out of 14 survivors misclassified.
